# Novel Missense and Splice Site Mutations in *USH2A*, *CDH23*, *PCDH15*, and *ADGRV1* Are Associated With Usher Syndrome in Lebanon

**DOI:** 10.3389/fgene.2022.864228

**Published:** 2022-05-16

**Authors:** Lama Jaffal, Hanane Akhdar, Hawraa Joumaa, Mariam Ibrahim, Zahraa Chhouri, Alexandre Assi, Charles Helou, Hane Lee, Go Hun Seo, Wissam H. Joumaa, Said El Shamieh

**Affiliations:** ^1^ Rammal Hassan Rammal Research Laboratory, PhyToxE Research Group, Faculty of Sciences, Lebanese University, Nabatieh, Lebanon; ^2^ Department of Biological and Chemical Sciences, School of Arts and Sciences, Lebanese International University, Beirut, Lebanon; ^3^ Retinal Service, Beirut Eye & ENT Specialist Hospital, Beirut, Lebanon; ^4^ Rare Genetic Disease Research Center, 3billion Inc, Seoul, South Korea; ^5^ Department of Medical Laboratory Technology, Faculty of Health Sciences, Beirut Arab University, Beirut, Lebanon

**Keywords:** Usher syndrome, mutation, genetics, developing countries, rare disease, next-generation sequencing

## Abstract

The purpose of this study was to expand the mutation spectrum by searching the causative mutations in nine Lebanese families with Usher syndrome (USH) using whole-exome sequencing. The pathogenicity of candidate mutations was first evaluated according to their frequency, conservation, and *in silico* prediction tools. Then, it was confirmed *via* Sanger sequencing, followed by segregation analysis. Finally, a meta-analysis was conducted to calculate the prevalence of USH genes in the Lebanese population. Three missense mutations, two splice site mutations, and one insertion/deletion were detected in eight of the families. Four of these variants were novel: c.5535C > A; p.(Asn1845Lys) in exon 41 of *CDH23*, c.7130G > A; p.(Arg2377Gln) in exon 32 of *ADGRV1*, c.11390-1G > A in *USH2A*, and c.3999–6A > G in *PCDH15*. All the identified mutations were shown to be likely disease-causing through our bioinformatics analysis and co-segregated with the USH phenotype. The mutations were classified according to the ACMG standards. Finally, our meta-analysis showed that the mutations in *ADGRV1*, *USH2A*, and *CLRN1* are the most prevalent and responsible for approximately 75% of USH cases in Lebanon. Of note, the frequency USH type 3 showed a relatively high incidence (23%) compared to the worldwide prevalence, which is around 2–4%. In conclusion, our study has broadened the mutational spectrum of USH and showed a high heterogeneity of this disease in the Lebanese population.

## Introduction

Usher syndrome (USH) is a genetic disorder combining various degrees of sensorineural hearing impairment with a gradual vision loss caused by retinitis pigmentosa (RP) (Boughman et al., 1983; Fuster-García et al., 2018; Castiglione and Möller, 2022). Hearing impairment is mainly due to changes in morphogenesis and stability of stereocilia projections on the cochlear hair cells that mediate sound transduction (Frolenkov et al., 2004; Millán et al., 2011). In addition, patients may suffer from balance defects due to disturbances in hair cells of the vestibular apparatus (Frolenkov et al., 2004; Millán et al., 2011). The visual loss driven by RP in USH patients starts with problems in night vision and reduced peripheral vision (Hartong et al., 2006). As the disease progresses, the central vision also begins to deteriorate, eventually leading to dysfunction and blindness (Edwards et al., 1998). The prevalence of USH ranges between 1 and 4 per 25,000 individuals among different populations, making it number 1 cause of deaf-blindness cases worldwide (Mathur and Yang, 2015) and in Lebanon (Saouda et al., 1998; Verpy et al., 2000; Audo et al., 2012; Reddy et al., 2014).

The clinical manifestation of most USH cases is divided into three distinct subtypes: type 1 (USH1), type 2 (USH2), and type 3 (USH3) (Petit, 2001; Mathur and Yang, 2015). The main differences between these clinical subtypes reside in the severity and progression of hearing impairment, the onset age of RP, and the implication of vestibular dysfunction or not (Petit, 2001; Mathur and Yang, 2015). USH3 is the rarest among these three subtypes as it accounts for only 2–4% of affected individuals (Yan and Liu, 2010). Moreover, some USH cases do not match the same clinical profile of any of these subtypes, and thus, they cannot be classified into any of them and are considered atypical USH forms (Liu et al., 1998; Mathur and Yang, 2015).

Today, mutations in sixteen genes have been associated with USH, all inherited in an autosomal recessive manner (RetNet: https://sph.uth.edu/retnet/). Noteworthy, some cases of digenic inheritance of USH were reported in the literature; for instance, *PDZD7* variants combined with *ADGRV1* (NG_007083) variants (Ebermann et al., 2010) and *CDH23* (NG_008835) variants combined with *PCDH15* (NG_009191) variants were also reported (Zheng et al., 2005). Herein, we aimed to expand the mutation spectrum by searching the causative genetic mutations in nine Lebanese USH families, using targeted and whole-exome sequencing (WES).

## Methods

### Ethics Statement and Clinical Examinations

The Institutional Review Board of Beirut Arab University approved the study (2017H-0030-HS-R-0208). Written informed consent was obtained from all patients, and all procedures adhered to the tenets of the Declaration of Helsinki. As previously described, the affected individuals underwent clinical ophthalmic examination at Beirut Eye and ENT Specialist Hospital (Beirut, Lebanon) (Jaffal et al., 2019).

### Mutational Screening

Genomic DNA was extracted from whole blood samples of the affected individuals and their family members using the QIAamp DNA Mini extraction kit (Qiagen, Hilden, Germany). Extracted DNA samples were quantified using a dsDNA HS Assay kit on a Qubit3.0 fluorometer (Thermo Fisher Scientific, ShahAlam, Malaysia). The DNA samples of all the index patients were investigated by whole-exome sequencing (WES) as previously described (Jaffal et al., 2019), except the sample of the index in family 13, which was analyzed using targeted NGS, as described elsewhere (Audo et al., 2012; Kikuchi et al., 2015).

### Data Analysis and Interpretation of the Genetic Mutations

To identify the top 10 candidate mutations in every index patient, we have searched the public genome databases, including Genome Aggregation Database (gnomAD) (Karczewski et al., 2019), Trans-Omics for Precision Medicine (TOPMed) program (Millán et al., 2011), Ensembl GRCh37 genome browser (Zerbino et al., 2018), and Single Nucleotide Polymorphism Database (dbSNP build 152) (https://www.ncbi.nlm.nih.gov/snp/). These databases were used to ﬁlter out common variants with a minor allele frequency (MAF) higher than 0.01. Among the remaining mutations, splice site, non-sense mutations, missense, and insertions/deletions (indels) were prioritized. For the pathogenicity assessment, the level of evolutionary conservation of the affected residues across diﬀerent species was checked using the genome browser of the University of California at Santa Cruz (UCSC) (Supplementary Figures S1–S3) (Kent et al., 2002). The RetNet database (https://sph.uth.edu/retnet/) was used to check the association of the mutated genes with USH and confirm the clinical diagnosis. PolyPhen-2 (Adzhubei et al., 2010), scale-invariant feature transform (SIFT) (Kumar et al., 2009), and MutationTaster (Schwarz et al., 2014) computational prediction programs were used to predict the possible clinical significance of the detected mutations. Human Gene Mutation Database (Stenson et al., 2014), Leiden Open Variation Database (Fokkema et al., 2005), PubMed (https://www.ncbi.nlm.nih.gov/pubmed/), and Online Mendelian Inheritance in Man (https://omim.org/) were used to check if the identified mutations were novel or previously reported. Approximately half of the samples showed one homozygous candidate mutation after the filtering approach (Supplementary Table S1).

### Polymerase Chain Reaction, Sanger Sequencing, and Co-Segregation Analysis

The candidate mutations detected by NGS were validated through conventional polymerase chain reaction (PCR) using a T100 thermal cycler (Biorad, Kaki Bukit, Singapore), followed by Sanger sequencing on a 3730xl DNA Sequencer (Applied Biosystems, Courtaboeuf, Les Ulis, France). For co-segregation analysis in available family members, DNA fragments harboring the mutations were amplified and then Sanger sequenced. The used primers are available upon request.

## Results

The available medical history and clinical findings of the patients are summarized in Table 1. Stringent filtration and analysis of NGS data revealed the presence of homozygous variants that are likely associated with USH in eight of the families (Table 2), while we were not able to find any likely causative variant in one family.

**TABLE 1:** Summary of medical history and clinical findings of Usher patients belonging to eight Lebanese families.

Family	F6	F13	F16	F16	F25	F25	F40	F42	F53	F54
Individual	II.1	II.2	II.1	III.1	II.1	II.2	II.2	II.4	II.1	II.3
Gender	Female	Male	Male	Male	Male	Female	Female	Male	Female	Male
Age (years)	54	37	42	27	23	15	53	17	24	12
Onset age of hearing loss	Congenital	Third decade	Third decade	First decade	Congenital	Congenital	Third decade	Congenital	Congenital	First decade
Onset age of RP (years)	First decade	Early 20s	∼25	∼7	∼16	∼10	∼17	∼5–6	∼12	∼5
Vestibular dysfunction	Occasional	Occasional	No	No	No	No	Occasional	No	Yes	Occasional
Full-ﬁeld electroretinogram (ERG)	NA	Reduced photopic and very reduced scotopic ERG	Very reduced photopic and scotopic ERG	Very reduced photopic and scotopic ERG	Very reduced photopic and scotopic ERG	Very reduced photopic and scotopic ERG	NA	NA	Very reduced photopic and scotopic ERG	NA
Fundus photography	NA	NA	Abnormal macular reflex with marked pigmentary changes around the vascular arcades	Widespread pigmentary and atrophic retinal changes with macular sparing	Abnormal reflex at the maculae with an attenuation in the caliber of retinal vessels	Abnormal reflex at the maculae with a mild attenuation in the caliber of retinal vessels	NA	Widespread and patchy pigmentary changes	NA	Abnormal reflex at both maculae with an attenuation in the caliber of retinal vessels
Fundus autoﬂuorescence imaging	NA		Rings of hyperfluorescence at both maculae	Hyperfluorescence at both maculae	Rings of hyperfluorescence at both maculae	Ring of hyperfluorescence at the right macula	NA	Hyperfluorescence at both maculae		No abnormality

F signifies family. NA signifies not available.

**TABLE 2:** Details of mutations identiﬁed in eight Lebanese USH families.

Family	Gene and reference sequence	Exon	rs id	Nucleotide exchange	Effect on protein	Frequencies	PolyPhen-2 (score)	SIFT	MutationTaster	Novel/reported
F6 and F42	*CDH23* NM_022124.6	41	-	c.5535C > A	p.(Asn1845Lys)	0 (gnomAD) 0 (TOPMed) Never Hom	Probably damaging (0.99)	Not tolerated	Deleterious	Novel
F13	*ADGRV1* NM_032119.4	32	rs369341309	c.7130G > A	p.(Arg2377Gln	0.000011334 (TOPMed) Never Hom	Probably damaging (0.99)	Not tolerated	Deleterious	Novel
F16 and F54	*CLRN1* NM_001195794.1	2	rs397517932	c.301_305delGTCAT	p.(Val101SerfsTer27)	0.000007956 (gnomAD) Never Hom			Deleterious	Previously reported by Akoury et al. (2011)
F25	*USH2A* NM_206,933.4	-	-	c.11390-1G > A	splicing error	0 (gnomAD) 0 (TOPMed) Never Hom			Deleterious	Novel
F40	*CLRN1* NM_001195794.1	1	-	c.188A > C	p.(Tyr63Ser)	0 (gnomAD) 0 (TOPMed) Never Hom	Probably damaging Boughman et al. (1983)	Not tolerated	Deleterious	Previously reported by Jaffal et al. (2019)
F53	*PCDH15* NM_001142763.2	-	-	c.3999-6A > G	splicing error	0 (gnomAD) 0 (TOPMed) Never Hom			Deleterious	Novel

**FIGURE 1:**
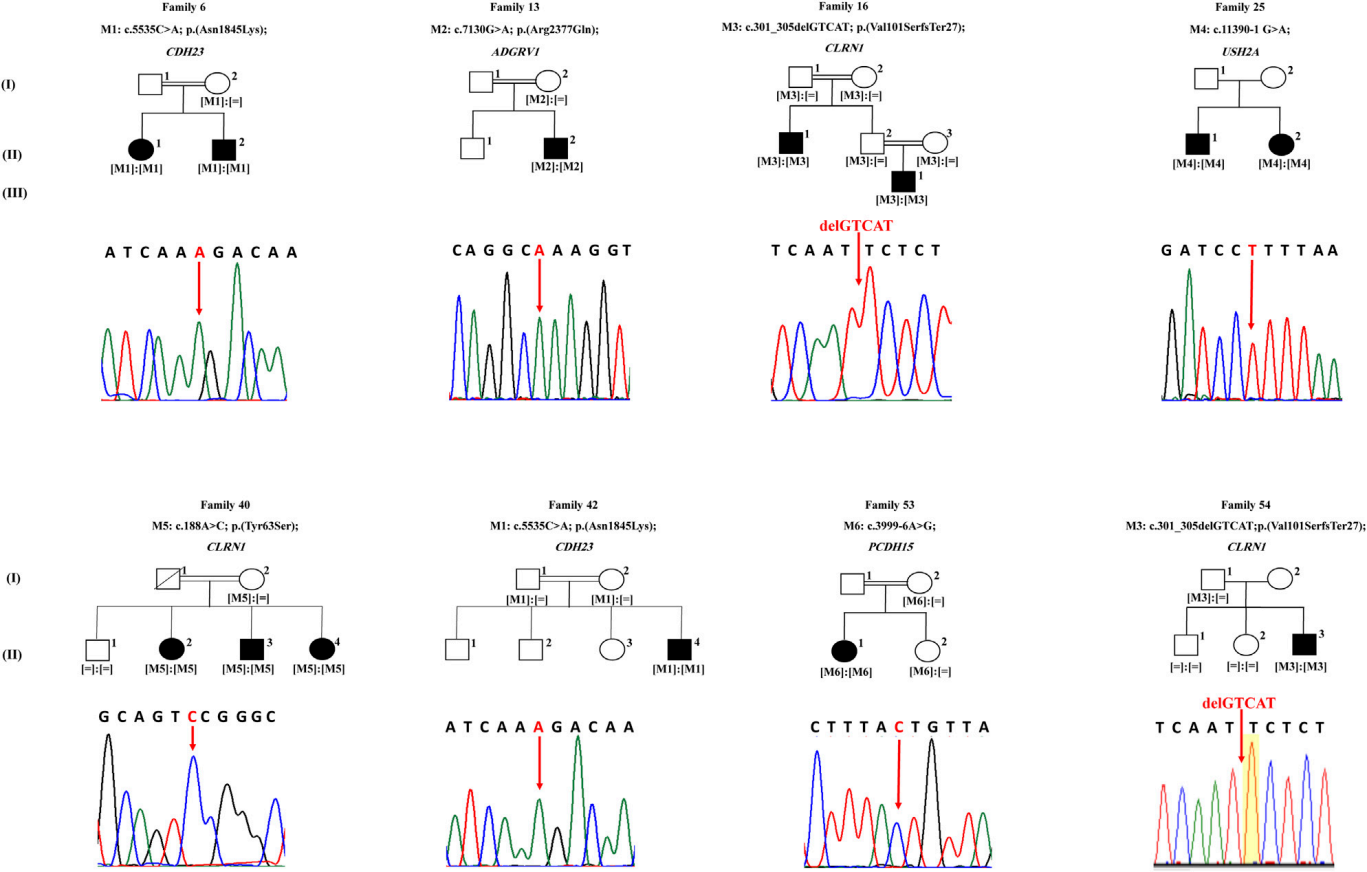
Pedigrees of eight families with Usher syndrome. Sequencing results of each proband are shown under the corresponding pedigree, noting that sequences of probands of families 25 and 53 are in reverse direction. White symbols indicate unaﬀected members. Black symbols indicate aﬀected members. Square and round symbols represent male and female individuals, respectively. The slash indicates deceased individuals. Double horizontal lines represent consanguineous unions. [M] deﬁnes mutated alleles. [ = ] defines wild type alleles.

In family 6, proband II.1 (a 54-year-old woman) suffered from a congenital profound hearing loss, with no intelligible speech, in addition to occasional dizziness and balance problems. She started experiencing RP symptoms during her first decade (Table 1). NGS showed that she carried the novel homozygous missense variant: c.5535C > A; p.(Asn1845Lys) in exon 41 of *CDH23* (Figure 1). According to UCSC, the Asn residue affected by this variant is highly conserved across species. The variant was absent in gnomAD and TOPMed populations (Table 2). It was also predicted to be probably damaging, not tolerated, and deleterious according to PolyPhen-2, SIFT, and MutationTaster, respectively (Table 2). Sanger sequencing showed that the affected sibling (II.2) also carried the variant in a homozygous state. At the same time, the unaffected mother (I.2) was a heterozygous carrier, indicating that the variant co-segregated well with the phenotype (Figure 1). According to the standards developed by the American College of Medical Genetics and Genomics (ACMG), this variant is classified as likely pathogenic (Supplementary Table S1).

The patient of family 13 (a 37 year old male) was initially diagnosed with RP around the age of 25, but later on, he started experiencing a mild hearing impairment within his third decade (Table 1). NGS revealed that he harbored a novel homozygous missense variant: c.7130G > A; p.(Arg2377Gln); rs369341309; in exon 32 of *ADGRV1* (Figure 1). According to the UCSC genome browser, the Arg residue affected by this variant is highly conserved. The variant was not detected in the gnomAD population, while it was shown to be very rare and never homozygous in the TOPMed population (0.000011334) (Table 2). Moreover, it was predicted to be probably damaging, not tolerated, and deleterious according to PolyPhen-2, SIFT, and MutationTaster, respectively (Table 2). The DNA sample of the unaﬀected mother (I.2) was screened and showed the candidate variant to be heterozygous in her, indicating that it co-segregated well with the phenotype (Figure 1). According to the standards of the ACMG, this variant is classified as having uncertain significance (Supplementary Table S1).

Family 16 comprised two affected members: proband II.1 (a 42-year-old man) and his nephew III.1 (a 27-year-old man) who started experiencing hearing impairment within their third and first decades, respectively. Both of them did not report balance problems. Patient II.1 has fully intelligible speech. Patient III.1 also has intelligible speech, although he presented speech difficulties that improved with speech therapy at a younger age. RP onset ages were around 27 years for patient II.2 and around 7 years for his nephew III.1 (Table 1). WES was carried in index patient II.1, who was found to have a homozygous frameshift deletion in exon 2 of *CLRN1* (NG_009168): c.301_305delGTCAT; p.(Val101SerfsTer27); rs397517932; resulting in the introduction of a premature termination codon, 27 amino acids downstream (Figure 1). Sanger sequencing showed that patient III.1 also carried this variant in the homozygous state. The variant was shown to be rare heterozygous in gnomAD (0.000007956), while it was not detected in TOPMed populations (Table 2). It co-segregated well with the phenotype as parents of both patients (I.1, I.2, II.2, and II.3) were shown to be heterozygous carriers (Figure 1). Interestingly, this 5-bp deletion was previously reported in another Lebanese USH family in 2011 (Akoury et al., 2011). According to the standards of the ACMG, this variant is classified as pathogenic (Supplementary Table S1).

Patients of family 25 (a 23-year-old man and a 15-year-old woman) suffered from a congenital hearing loss, with no balance problems. They started experiencing RP symptoms around 16 and 10, respectively (Table 1). WES was carried in the proband II.1 and showed her to harbor the homozygous acceptor splice site variant: c.11390-1G > A in *USH2A* (NG_009497) (Figure 1). Sanger sequencing revealed that his affected sister (II.2) also carried the variant in a homozygous state (Figure 1). The variant was not found in gnomAD nor TOPMed populations (Table 2). According to MutationTaster, it was predicted to be deleterious (Table 2). No DNA samples of additional family members were available for co-segregation analysis. According to the standards of the ACMG, this variant is classified as pathogenic (Supplementary Table S1).

Proband II.2 of family 40 (a 53-year-old woman) started experiencing RP symptoms around 17 years, while hearing impairment started in her third decade. She also reported experiencing occasional balance problems (Table 1). WES showed her to carry a missense homozygous variant c.188A > C: p.(Tyr63Ser) in exon 1 of *CLRN1* (Figure 1). This variant aﬀects a highly conserved amino acid according to UCSC. It is absent in gnomAD and TOPMed populations and predicted to be probably damaging, not tolerated, and deleterious according to PolyPhen-2, SIFT, and MutationTaster, respectively (Table 2). Sanger sequencing showed that her affected siblings (II.3 and II.4) also carried the variant in a homozygous state, while the unaﬀected mother (I.2) was a heterozygous carrier. At the same time, the unaffected sibling (II.1) did not carry it, indicating that it co-segregated well with the phenotype (Figure 1). Of note that we previously detected this same *CLRN1* variant in another Lebanese USH patient in 2019 (Jaffal et al., 2019). According to the standards of the ACMG, this variant is classified as likely pathogenic (Supplementary Table S1).

Patient II.1 of family 42 (a 17-year-old boy) suffered from a congenital profound hearing loss, with no intelligible speech at all. No balance problems were reported. He started experiencing RP symptoms around the age of 5. NGS showed him to carry the same *CDH23* homozygous variant that is detected in patients of family 6: the c.5535C > A; p(Asn1845Lys) in exon 41 (Figure 1; Table 2). It co-segregated well with the phenotype as both parents (I.1 and I.2) were shown to be heterozygous carriers (Figure 1). According to the standards of the ACMG, this variant is classified as likely pathogenic (Supplementary Table S1).

The patient of family 53 (a 24-year-old woman) suffered from a congenital profound hearing loss, with no intelligible speech, in addition to frequent dizziness and disequilibrium. She started experiencing RP symptoms around the age of 12. WES showed her to carry the homozygous splice region variant c.3999--6A > G in *PCDH15* (Figure 1). The variant was not found in gnomAD nor TOPMed populations (Table 2). According to MutationTaster, it was predicted to be deleterious (Table 2). We have tested this variation by splice AI, and it showed a probability of 62 and 99% to affect the −6 and −1 positions of the pre-mRNA. It co-segregated well with the phenotype as the unaffected mother (I.2) and sister (II.2) were shown to be heterozygous carriers (Figure 1). According to the standards of the ACMG, this variant is classified as having uncertain significance (Supplementary Table S1).

The patient of family 54 (a 12-year-old boy) started experiencing both hearing impairment and RP symptoms in his first decade. He also shares occasional balance problems (Table 1). WES showed him to carry the same *CLRN1* homozygous variant that was detected in patients of family 16: c.301_305delGTCAT; p.(Val101SerfsTer27) (Figure 1; Table 2). Sanger sequencing showed that the unaffected mother (I.2) was a heterozygous carrier, while both unaffected siblings (II.1 and II.2) were wild type, indicating that the variant co-segregated well with the phenotype (Figure 1). According to the standards of the ACMG, this variant is classified as pathogenic (Supplementary Table S1).

**FIGURE 2:**
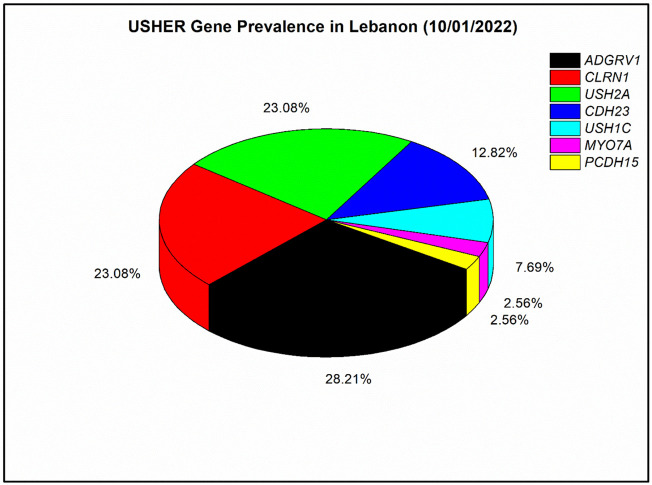
Prevalence of mutated Usher genes detected in the Lebanese population to date (till 10/01/2022). A meta-analysis with four previously published articles was done (Verpy et al., 2000; Akoury et al., 2011; Reddy et al., 2014; Jaffal et al., 2019); in total, 39 affected individuals with Usher syndrome were included. The prevalence analysis is based on the number of affected individuals and not families as in Reddy et al. (2014).

In our nine solved families, each of *ADGRV1* and *PCDH15* were responsible for USH in one patient, *USH2A* was responsible for USH in two patients, *CDH23* was responsible for USH in three patients, and *CLRN1* was responsible for USH in seven patients*.* In addition, ten other Lebanese families were genetically screened in previous studies (Saouda et al., 1998; Verpy et al., 2000; Akoury et al., 2011; Reddy et al., 2014). In those ten families, *MYO7A* was responsible for USH in one patient, each of *CDH23* and *CLRN1* were responsible for USH in two patients, *USH1C* was found mutated in three patients, *USH2A* was found mutated in seven patients, and *ADGRV1* was found mutated in ten patients. Overall, seven USH genes have been responsible for USH in the 39 Lebanese affected individuals published so far (Figure 2). Mutations in *ADGRV1* (28%), *USH2A* (23%), and *CLRN1* (23%) were the most prevalent. *CDH23* followed with 13%, while *PCDH15*, *USH1C*, and *MYO7A* were the least prevalent, with a frequency ranging from ∼8 to 3% (Figure 2).

## Discussion

This study used mainly WES to determine the likely causative mutations in nine Lebanese families with a presumed diagnosis with USH. Extensive bioinformatics analysis of NGS data revealed the presence of six homozygous mutations in eight families, while the case of one family remained unsolved. Of note, consanguineous marriages were present in six out of the eight solved families (Figure 1). Of the detected variants, four were novel (Table 2). Those variants were shown to be heterozygous or absent in the available unaﬀected family members, indicating that they co-segregate adequately with the USH phenotype (Figure 1).

In two of the families (Hartong et al., 2006; Jouret et al., 2019), the patients were shown to carry the same homozygous variant in *CDH23* that encodes for cadherin 23, a protein needed to establish and/or maintain the proper growth and excellent cohesion of the hair bundle in the vestibular organ and the cochlea (El-Amraoui and Petit, 2005; Pan and Zhang, 2012). *CDH23* is known to cause USH1 characterized by a congenital severe-to-profound hearing loss, vestibular dysfunction, and early onset of RP within the ﬁrst decade, making it the most severe subtype (Petit, 2001; Mathur and Yang, 2015). The clinical profiles of the patients in these two families are in favor of USH1, since they suffer from a severe congenital hearing impairment with no intelligible speech at all (Table 1). The patients of family 6 also have balance problems. However, the patient of family 42 did not experience balance problems to date, keeping in mind that this patient is still young (17 years) and might develop vestibular dysfunction at an older age.

Patient II.2 of family 13 was initially diagnosed with RP in his early twenties. Still, genetic testing showed that he harbored a homozygous variant in *ADGRV1*, a gene related to USH and previously known as *GPR98*. Consequently, we re-investigated his clinical history and queried if he suffered from additional symptoms other than the visual impairment. Indeed, this revealed that he was experiencing a mild, stable hearing impairment and occasional dizziness that developed within his third decade, which caused him to use hearing aids. Thus, this led to a revised re-diagnosis with USH. This observation highlights the importance of genetic testing in getting an accurate clinical diagnosis, especially in syndromic inherited retinal diseases that can be misdiagnosed because the systemic symptoms may only appear late in life, or because ophthalmologists may only focus on the visual impairment without considering other symptoms and relating them to the case.

*ADGRV1* encodes the adhesion of G-protein-coupled receptor V1 that is needed for the normal development and maintenance of auditory hair bundles of the cochlear sensory hair cells (McGee et al., 2006). It is usually known to cause USH2 characterized by congenital moderate-to-severe hearing loss, absence of vestibular dysfunction, and RP manifestation from the second decade onward (Petit, 2001; Weston et al., 2004). However, the clinical profile of this patient was compatible with neither USH2 features nor any of the other classical subtypes, since he reported a late mild hearing impairment that only appeared in adulthood and had an intelligible speech. This confirms that the hearing impairment occurrence was post-lingual and not congenital, unlike the cases of USH1 and USH2. The patient also stated that his hearing impairment is stable and not progressing over the years, in contrast to the hearing impairment in USH3 that is characterized by its progressiveness. Accordingly, this case may be considered an atypical USH form.

The affected members in families 16, 40, and 54 were shown to carry homozygous *CLRN1* mutations. The clarin-1 protein encoded by this gene is involved in the morphogenesis of the hair bundle and the synaptic transmission between light-sensitive photoreceptor cells and their associated neurons, in addition to being potentially engaged in the maintenance and development of stereocilia (Zallocchi et al., 2009; Dulon et al., 2018). *CLRN1* variants are usually associated with USH3, which is characterized by post-lingual hearing impairment with a progressive nature, variable vestibular dysfunction, and variable onset of RP (Audo et al., 2012; Jaffal et al., 2019). Patients of families 16 and 40 had intelligible speech, confirming the post-lingual hearing loss. They also reported that their hearing impairment is progressive and worsens over the years, which is in accordance with USH3. On the contrary, the patient of family 54 had a prelingual hearing impairment diagnosed as early as the age of 1, which is not in accordance with USH3.

We previously reported another Lebanese USH3 family having the same missense mutation in *CLRN1;* p(Tyr63Ser) (Jaffal et al., 2019). In addition, a Lebanese family comprising two USH patients, with one of them presenting symptoms of USH3, was reported by Akoury et al. (2011). Interestingly, the latter two USH patients carried the same *CLRN1* 5-bp deletion that is identified in families 16 and 54 in this study. This suggests that these two mutations might have a founder effect in the Lebanese population. Of note, most of CLRN1-affected individuals (from the current and previous studies) come from the south region of Lebanon.

Patients of family 25 were shown to carry a homozygous acceptor splice site variant in *USH2A* encoding for Usherin, which is needed for retinal photoreceptor retention and normal cochlear hair cell growth (Liu et al., 2007). Disruption of this same splice site has been observed in a Caucasian USH family (but c.11390-1G > C instead of c.11390-1G > A) (Le Quesne Stabej et al., 2012). Acceptor and donor splice site variants usually result in a loss of protein function (Baralle and Baralle, 2005). *USH2A* mutations are the major cause of USH2 cases (Toualbi et al., 2020). USH2 description is in accordance with the clinical profile in the patients of this family who suffer from a congenital hearing impairment and RP manifestation from the second decade, with no vestibular dysfunction (Table 1).

The patient of family 53 carries the homozygous splice region variant c.3999-6A > G in *PCDH15*. PCDH15 is expressed in the neurosensory epithelium of the eye and ear to maintain the function of stereocilia and retinal photoreceptor cells (Ahmed et al., 2003). *PCDH15* mutations are known to cause USH1 (Ahmed et al., 2003), which is in accordance with the clinical profile showing a severe congenital prelingual hearing impairment and vestibular dysfunction (Table 1).

To date, we were able to genetically screen 10 Lebanese USH families: one in a previous study (Jaffal et al., 2019) and nine in the current study; nine of those families were genetically solved, while one family remained unsolved. Therefore, until today, our success rate with USH families is 90%. This rate is similar to the diagnosis rates of USH in larger populations (77–93%) (Bonnet et al., 2016; Sun et al., 2018), which were also achieved using WES, proving this technique to be highly efficient in USH molecular diagnosis. Nevertheless, the use of whole-genome sequencing (WGS) might be efficient in improving those rates by identifying non-coding variants that WES and other standard sequencing techniques usually miss. For instance, a deep-intronic variant that escaped detection by panel-NGS, genome-wide linkage analysis, and WES was identified in CLRN1 (c.254–649T > G) using WGS (Bonnet et al., 2016). However, the high cost, the challenging filtration of extensive data, and the difficulty of proving the pathogenicity of non-coding variants are all factors that hinder WGS from being used as a routine diagnostic approach (Bonnet et al., 2016).

Our prevalence analysis shows heterogeneity in the genetics of USH among different populations. For instance, *ADGRV1*, which ranked first in Lebanese patients (Figure 2), is not usually among the most prevalent genes in other populations. A meta-analysis from 2019 showed it accounts for 5% of USH cases (Jouret et al., 2019); similarly, a recent study of a large Italian cohort also showed it accounts for 5% (Colombo et al., 2021). On the other hand, *USH2A* mutations usually show the highest prevalence in most other populations, such as previously reported Chinese, French, Italian, and Spanish cohorts (Fuster-García et al., 2018; Sun et al., 2018; Colombo et al., 2021; Mansard et al., 2021). Moreover, the relatively high prevalence of *CLRN1* variants (23%) suggests that USH3 may have a high prevalence in the Lebanese population, while this subtype is the rarest among the three USH subtypes as it accounts for only 2–4% of all cases in most populations (Audo et al., 2012). Exceptionally, the frequency of USH3 is significant in the Finnish population (40%) (Ness et al., 2003), the Ashkenazi Jews (40%) (Pakarinen et al., 1995), the population of Birmingham, United Kingdom (20%) (Edwards et al., 1998), and now in the Lebanese population (23%), specifically in the south region, from where our *CLRN1* patients originated. The prevalence of *CDH23* was around 13% in this analysis (Hope et al., 1997), while it ranged between 2 and 8% in other countries (Fuster-García et al., 2018; Jouret et al., 2019; Colombo et al., 2021; Wafa et al., 2021). Regarding *PCDH15* and *USH1C*, there was no significant difference with other cohorts. In contrast, *MYO7A*, which is among the least frequent genes in this analysis (around 3%), is detected in much higher frequencies in most other populations and considered as a major USH gene (Fuster-García et al., 2018; Sun et al., 2018; Colombo et al., 2021; Mansard et al., 2021).

In conclusion, the results of this study have broadened the mutational spectrum of USH and showed a high heterogeneity of this disease in the Lebanese population. The screening of more Lebanese USH families would be essential to elucidate the genetic characteristics of USH and the accurate prevalence of its three subtypes and causative genes in this population.

## Data Availability

The original contributions presented in the study are publicly available. These data can be found at: SCV002097249—SCV002097254.
