# Supplementary material for: Novel Missense and Splice Site Mutations in USH2A, CDH23, PCDH15, and ADGRV1 Are Associated With Usher Syndrome in Lebanon
Source: Front Genet. 2022 May 16;13:864228. doi: 10.3389/fgene.2022.864228 (PMC9149366; doi:10.3389/fgene.2022.864228)
Supplement: Supplementary file 2 [file DataSheet1.docx]

**Figure S1:** Conservation analysis for the c.5535C>A; p.(Asn1845Lys) in *CDH23*.


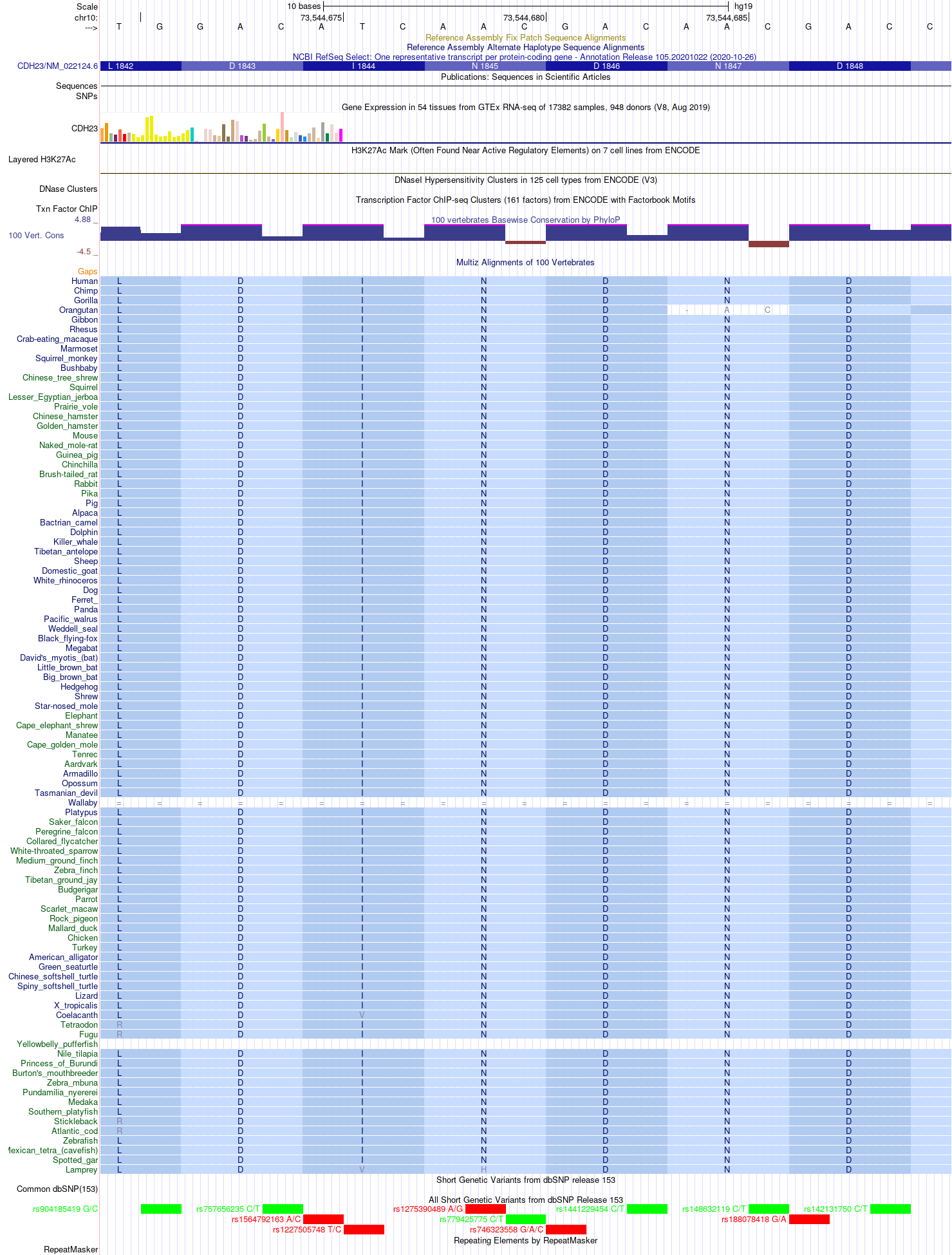


The level of evolutionary conservation of the affected residues across 100 diﬀerent species was checked using the genome browser of the University of California at Santa Cruz (GRCh37/hg19).

**Figure S2:** Conservation analysis for the c.7130G>A; p.(Arg2377Gln) in *ADGRV1*.


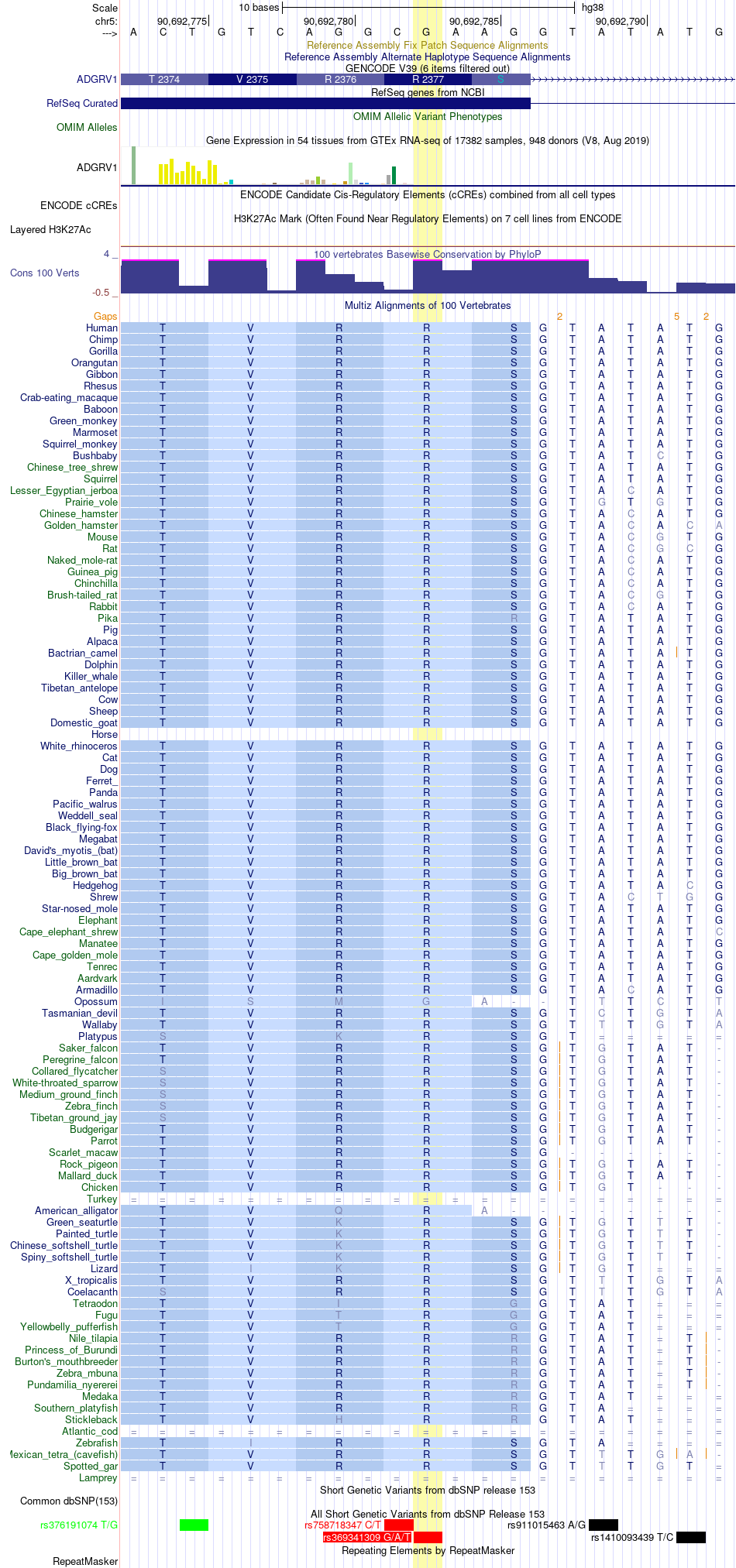


The level of evolutionary conservation of the affected residues across 100 diﬀerent species was checked using the genome browser of the University of California at Santa Cruz (GRCh37/hg19).

**Figure S2:** Conservation analysis for the c.188A>C; p.(Tyr63Ser) in *CLRN1*.


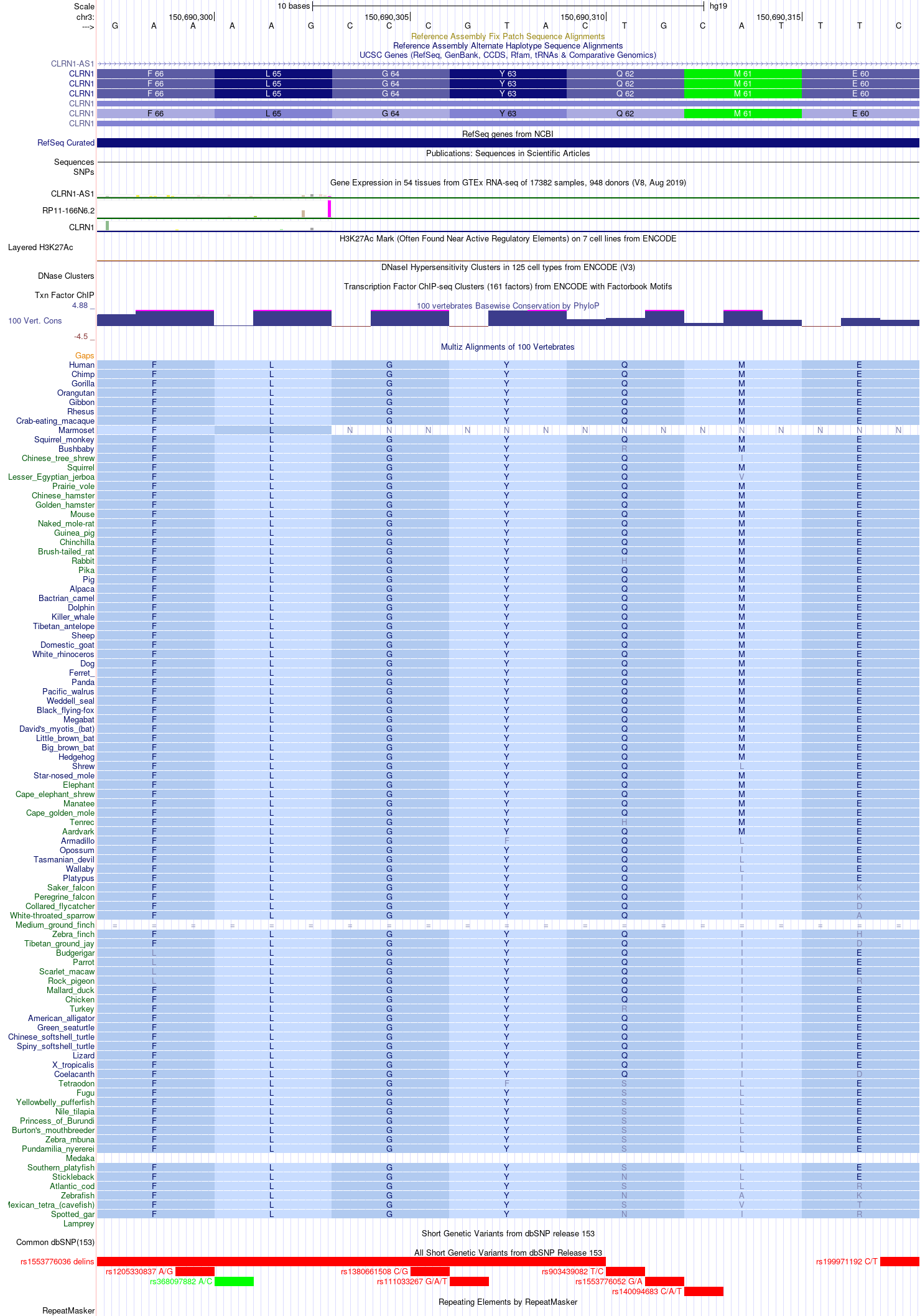


The level of evolutionary conservation of the affected residues across 100 diﬀerent species was checked using the genome browser of the University of California at Santa Cruz (GRCh37/hg19).
